# RNA demethylase ALKBH5 promotes tumorigenesis of t (8;21) acute myeloid leukemia via ITPA m6A modification

**DOI:** 10.1186/s40364-023-00464-x

**Published:** 2023-03-10

**Authors:** Ran Li, Xiaolu Wu, Kai Xue, Dandan Feng, Jianyong Li, Junmin Li

**Affiliations:** 1 Shanghai Institute of Hematology, State Key Laboratory of Medical Genomics, National Research Center for Translational Medicine at Shanghai, Ruijin Hospital, Shanghai Jiao Tong University School of Medicine, Shanghai, China; 2 Department of Hematology, Jiangsu Province Hospital, The First Affiliated Hospital of Nanjing Medical University, Jiangsu Key Lab of Cancer Biomarkers, Prevention and Treatment, Collaborative Innovation Center for Personalized Cancer Medicine, Nanjing Medical University, Nanjing, China; 3 Department of Child Health Care, Nanjing Maternity and Child Health Care Hospital, Women’s Hospital of Nanjing Medical University, Nanjing, China; 4 Department of Pediatrics, The First Affiliated Hospital, Nanjing Medical University, Nanjing, China

**Keywords:** ALKBH5, m6A methylation, Leukemia stem/initiating cells, t (8;21) acute myeloid leukemia, Transcription factors

## Abstract

**Background:**

Although t (8;21) is in fact considered a good risk acute myeloid leukemia (AML), only 60% of the patients live beyond 5 years after diagnosis. Studies have shown that RNA demethylase ALKBH5 promotes leukemogenesis. However, the molecular mechanism and clinical significance of ALKBH5 in t (8;21) AML have not been elucidated.

**Methods:**

The expression of ALKBH5 was assessed in t (8;21) AML patients via qRT-PCR and western blot. The proliferative activity of these cells was examined through CCK-8 or colony-forming assays, while flow cytometry approaches were used to examine apoptotic cell rates. The in vivo role of ALKBH5 promoting leukemogenesis was assessed using t (8;21) murine model, CDX, and PDX models. RNA sequencing, m6A RNA methylation assay, RNA immunoprecipitation, and luciferase reporter assay were used to explore the molecular mechanism of ALKBH5 in t (8;21) AML.

**Results:**

ALKBH5 is highly expressed in t (8;21) AML patients. Silencing ALKBH5 suppresses the proliferation and promotes the apoptosis of patient-derived AML cells and Kasumi-1 cells. With integrated transcriptome analysis and wet-lab confirmation, we found that ITPA is a functionally important target of ALKBH5. Mechanistically, ALKBH5 demethylates ITPA mRNA and increases its mRNA stability, leading to enhanced ITPA expression. Furthermore, transcription factor TCF15, specifically expressed in leukemia stem/initiating cells (LSCs/LICs), is responsible for the dysregulated expression of ALKBH5 in t (8;21) AML.

**Conclusion:**

Our work uncovers a critical function for the TCF15/ALKBH5/ITPA axis and provides insights into the vital roles of m6A methylation in t (8;21) AML.

**Supplementary Information:**

The online version contains supplementary material available at 10.1186/s40364-023-00464-x.

## Introduction

The balanced chromosomal translocation t (8;21) is one of the most common genetic aberrations in acute myeloid leukemia (AML), and 40–50% of patients with t (8;21) inevitably relapse treated with chemotherapy alone, even though it is considered as favorable-risk AML [1, 2]. KIT mutations appear to be the most common additional genetic events in the t (8;21) patients and show a poor prognosis [3]. Allogeneic hematopoietic stem cell transplantation (alloHSCT) seems to be a curative approach for patients with KIT mutations. Unfortunately, even after HSCT, the 2-year cumulative incidence of relapse could approach 32%, and the 2-year leukemia-free survival was only 55% [4], indicating there is an urgent need to develop more effective therapeutic approaches to improve the patient’s prognosis.

N6-methyladenosine (m6A) is one of the most abundant modifications in mRNA and is reversible and dynamically regulated by methyltransferases (writers), and demethylases (erasers) [5]. AlkB homolog 5 (ALKBH5) has been found commonly dysregulated in multiple malignancies and plays a vital role as an m6A demethylase [6]. ALKBH5 is upregulated in breast cancer and plays an oncogenic role via promoting proliferation and migration [7]. ALKBH5 knockout in lung adenocarcinoma cells decreases FOXM1 translation by elevating its m6A abundance and reducing cell proliferation and invasion [8]. ALKBH5, but not METTL3, inhibits cell adhesion of bladder cancer by repressing ITGA6 protein synthesis mediated by YTHDF1 and YTHDF3 [9]. ALKBH5 exerts tumor-suppressive effects by reducing WIF1 mRNA methylation and modulating the Wnt pathway, sensitizing pancreatic ductal adenocarcinoma cells to gemcitabine [10]. Hence, dysregulated ALKBH5 expression could promote or suppress carcinogenesis based on cancer types.

It is widely accepted that LSCs acquire aberrant self-renewal capacity in contrast to normal HSPCs with restricted self-renewal capacity, which contributes to the leukemogenesis and relapse of AML. One important regulator of the survival and growth of LSCs appears to be the dysregulation of the transcription factor [11]. Transcriptional deregulation leads to leukemogenesis and is crucially involved in the.

pathogenesis of AML [12]. TCF15 (also known as Paraxis) is a known transcription factor, suggesting a potential regulatory role in the molecular program controlling HSC output. TCF15 plays an important role in pluripotency exit, somitogenesis, paraxial mesoderm development, HSC quiescence, and long-term self-renewal [13–16]. In hematopoiesis, Tcf15 overexpression inhibited HSC proliferation in vitro and led to the inhibition of hematopoietic differentiation in stably reconstituted mice [16]. However, the role of TCF15 in AML is still unclear.

Previous studies reported that ALKBH5 plays a critical role in promoting leukemogenesis via regulating TACC3 or AXL but exhibits little effect on normal hematopoiesis [17, 18]. However, according to our RNA-seq results, we found both TACC3 and AXL expression had no significant difference upon ALKBH5 knockdown in t (8;21) Kasumi-1 cells. Based on the high heterogeneity of the clinical biology of AML, we speculated that ALKBH5 might have unique mechanisms of pro-leukemogenesis in t (8;21) AML patients. We next performed a series of functional and mechanistic studies, which revealed that ALKBH5 plays a critical role in promoting leukemogenesis and LSC/LIC self-renewal as an m6A demethylase by post-transcriptional regulation of ITPA. In addition, previous studies failed to at the transcriptional level explain why ALKBH5 is highly expressed in AML. We found that the transcription factor TCF15 is responsible for the abnormal upregulated ALKBH5 expression in t (8;21) LSCs/LICs. These data highlight the TCF15/ALKBH5/ITPA axis as a promising target in t (8;21) AML treatment.

## Materials and methods

### Cell culture

Kasumi-1 cells were obtained from America Type Culture Collection (ATCC) and cultured in RPMI-1640 (Gibco) supplemented with 10% fetal bovine serum (Gibco) supplemented with 1% penicillin-streptomycin (Invitrogen) at 5% CO_2_ and 37 °C incubators. Primary murine BM cells were cultured in DMEM (Gibco) with 15% fetal bovine serum (Gibco), stem cell factor, IL-3, IL-6, 1% penicillin-streptomycin (Invitrogen), and 5% WEHI-3B conditional medium. Primary patient cells were cultured in IMDM (Gibco) supplemented with 20% FBS (Gibco), 10 ng/ml human cytokines SCF, TPO, FLT3L, IL-3, and IL-6. All cytokines were obtained from Peprotech.

### Mice studies

The t (8;21) leukemia murine model carrying RUNX1-RUNX1T1 and KIT^N822K^ mutation was constructed as previous description [19]. For evaluating the role of ALKBH5 in AML maintenance, leukemic BM cells (RFP^+^) isolated from primary RUNX1-RUNX1T1 and KIT^N822K^ leukemia mice were transduced with shNC or shAlkbh5 and 1 × 10^6^ transduced cells were transplanted into lethally irradiated 6- to 8-week-old BALB/c recipient mice. The xenograft mouse model was established by injecting 1 × 10^6^ Kasumi-1 cells expressing shNC or shALKBH5 into NOD scid gamma (NSG) mice via tail vein. For the human AML PDX models, 1 × 10^6^ t (8;21) AML patient-derived bone marrow mononuclear cells (BMMNCs) expressing shNC or shALKBH5 were transplanted into NSG recipient mice intravenously. CO_2_ inhalation was used to end the lives of leukemic mice when they showed signs of systemic illness. Peripheral blood (PB) was collected for the complete blood count test. We isolated BM cells from both tibias and femurs and resuspended 100,000 of them in 200 mL of cold MACS buffer (1xPBS supplemented with 2 mmol/L EDTA and 0.5% BSA) and loaded them for cytospin preparation. After the leukemic mice were sacrificed, parts of their spleens and livers were collected, fixed in formalin, and embedded in paraffin. All experiments on mice in our research protocol were approved by the Institutional Animal Care and Use Committee of Ruijin Hospital affiliated with Shanghai Jiao Tong University School of Medicine.

### Primary samples

12 t (8;21) AML samples collected in this study were diagnosed in Shanghai Ruijin Hospital. All patients provided informed consent for sample collection. Protocols for bone marrow sample handling and data analysis were approved by the Institutional Review Board from Ruijin Hospital affiliated with Shanghai Jiao Tong University School of Medicine and were performed in compliance with the Declaration of Helsinki. Patient information is shown in Table S1. Normal human BMMNCs were obtained commercially from Lonza (Walkersville, Maryland).

### Transfection

ALKBH5 expression plasmid was constructed by cloning the full-length ORF of the human ALKBH5 gene into the pCDH lentiviral vector (GenePharma). ALKBH5 H204A was generated by GenePharma. The shRNA specifically targeting ALKBH5, Alkbh5, ITPA, TCF15, and the non-targeting control were purchased from GenePharma, China. Lentivirus packaging cells were transfected with LV3-pGLV-h1-GFPpuro vector (GenePharma) containing the shRNA above. The virus particles were harvested at 48 and 72 hours after transfection. For infection, cells were spinoculated with lentiviral supernatant supplemented with 5 μg/ml Polybrene (GenePharma) at 37 °C, 2000 rpm × 90 min for 2 consecutive days. The positive infected cells were selected with 1μg/ml puromycin (Sigma-Aldrich).

### Cell proliferation and apoptosis assays

CCK-8 proliferation assay kit (Dojindo, Japan) was used to detect cell viability. Cells were seeded at the concentration of 2000/well in a 96-wells plate. Cells were incubated at standard conditions for 1 h after 10 μl CCK-8 reagent was added and then the absorbance at 450 nm was measured with a microplate reader (Bio-Rad Laboratories, USA). For apoptosis assays, co-staining with Annexin V and PI allows discrimination among live cells (Annexin V-, PI-), early apoptotic cells (Annexin V+, PI-), and late apoptotic cells (Annexin V+, PI+). Cells transfected as described earlier were harvested using cold PBS/0.02% EDTA and washed twice in PBS. Double staining with FITC-Annexin V and Propidiumiodide (PI) was carried out using the Annexin V-FITC/PI apoptosis Detection Kit (KGA108, KeyGEN BioTECH) according to the manufacturer’s recommendations and then analyzed by FACS (BD BioSciences).

### Colony-forming assays

Cells were seeded into MethoCult H4434 Classic medium (StemCell Technologies) with the addition of 2.5 μg/ml puromycin. Cultures were incubated at 37 °C in a humidified atmosphere of 5% CO_2_. The colonies were replated every 7 days under the same conditions.

### Flow cytometric analysis

The BM of transplanted mice was harvested for the analysis of immunophenotypes. We stained cells at 4 °C for 30 min with various antibodies diluted in Flow Cytometry Staining Buffer (eBioscience) after blocking nonspecific binding with affinity-purified anti-mouse CD16/32 (eBioscience). After being resuspended in IC Fixation Buffer (eBioscience), cells are loaded for flow cytometry analysis in BD FACS FortessaX-20.

### Western blot

We used the Total Protein Extraction Kit (Keygentec, China) to extract cells or frozen tissues. BCA Protein Assay kit (Pierce, USA) was used to calculate the protein concentration. Western blot analysis followed a standard procedure. The primary antibodies (ALKBH5, ITPA, GAPDH, and β-actin) were obtained from Abcam, USA. The anti-mouse and anti-rabbit secondary antibodies were obtained from Cell Signaling Technology, USA.

### Quantitative real-time PCR (qRT-PCR)

We extracted total RNA with TRIzol (Invitrogen, USA) and synthesized cDNA with Primescript RT Reagent (Takara, Japan). SYBR®Premix Ex Taq™ Reagent (Takara, Japan) was used in the qRT-PCR. The relative expression was normalized using GAPDH, and the 2^−ΔΔCt^ method was applied to analyze the qRT-PCR results. The primers used for qRT-PCR were shown in Table S2.

### RNA sequencing

The cDNA library construction, sequencing, and transcriptome data analysis were performed by Personalbio Biotechnology Co., Ltd. (Shanghai, China). RNA-seq data used in this study have been deposited in the National Center for Biotechnology Information Sequence Red Archive (SRA) under the accession code SRR17828461-SRR17828472.

### m6A RNA Methylation Assay

The total RNA was detected using Abcam’s m6A RNA methylation assay kit. Briefly, RNAs of 400 ng were coated on the wells of the kit and incubated at 37 °C for 90 min. 50 μL capture antibody solution and 50 μL detection antibody solution were separately added to the wells and incubated for 60 and 30 minutes separately at room temperature. 450 nm wavelength was used on a microplate reader to determine the m6A level.

### RNA immunoprecipitation (RIP)

RIP assay was performed using the Magna RIP™ kit (Millipore). About 1 × 10^7^cells per sample were harvested and lysed in RIP lysis buffer. Anti-ALKBH5 or control IgG was incubated with magnetic beads for 2 h at 4 °C. The supernatant of RIP lysate, mixed with RIP buffer (Sigma-Aldrich), was added to the bead-antibody complexes for incubation overnight at 4 °C. Then, the beads were incubated with proteinase K buffer for 30 min at 55 °C, and RNA was finally extracted for qRT-PCR analysis.

### Gene-specific m6A qPCR

To detect m6A modifications on specific genes, we used the Magna MeRIP m6A Kit (Millipore) according to the manufacturer’s instructions. Briefly, a total of 200 mg of total RNA was broken down to approximately 100 nt in length by metal-ion induced fragmentation, purified, then incubated with an m6A antibody. The m6A IP portion was eluted twice by 100 ml competitively binding free m6A and recovered using the RNeasy kit (QIAGEN) after four washes in the IP buffer. We saved 10% of fragmented RNA as input controls and analyzed them by qPCR in conjunction with the MeRIP-ed RNAs. The fold enrichment of the anti-m6A antibody over the negative control mouse IgG was calculated as follows: Fold enrichment = 2^−ΔΔCt^.

### RNA stability assays

Human AML cells with or without ALKBH5 knockdown were exposed to actinomycin D (Sigma-Aldrich) at a final concentration of 5 μg/ml and collected at indicated time points. ITPA mRNA was analyzed by qRT-PCR.

### Luciferase reporter assays

Wild-type ALKBH5, TCF15 binding sites mutation ALKBH5, and wild-type ITPA plasmids were synthesized by GeneScript and cloned into the luciferase expression plasmid pGL3-Basic (Promega, USA). 100 ng luciferase reporter plasmids and 4 ng pRL-TK plasmid were transfected into 293 T cells using Lipofectamine™ 3000 (Invitrogen). Luciferase assay was conducted by a Dual-luciferase Reporter Assay System (Promega, USA). Luciferase activity was normalized to the activity of pRL-TK.

### Bioinformatics and statistical analyses

The “limma” package from R software was used to identify DEGs between shNC and shALKBH5 groups with appropriate filtering conditions (*p* < 0.05, |log_2_ FC| > 1.5). The “clusterProfiler” R package was used to perform GO and Kyoto Encyclopedia of Genes and Genomes (KEGG) analyses of these DEGs.

A global signal transduction network was constructed using Cytoscape V3.7.2 based on the KEGG database to reveal the inter-gene signaling of DEGs and identify core genes in this network. The nodes and lines in the network represented genes and interactions between the genes, respectively. The hub gene was selected based on the degrees. Genes with higher degrees have more crucial positions in the network.

All data are represented by mean ± standard deviation (SD) from three independent experiments. The R software (version 4.1.1, https://www.r-project.org/) was used to perform all bioinformatics and statistical analyses. The significance of data is estimated by Student’s t-test One-way ANOVA or Two-way ANOVA with *p* < 0.05.

## Results

### ALKBH5 is overexpressed in t (8;21) AML patients and is required for the growth of Kasunmi-1 cells

**Fig. 1:**
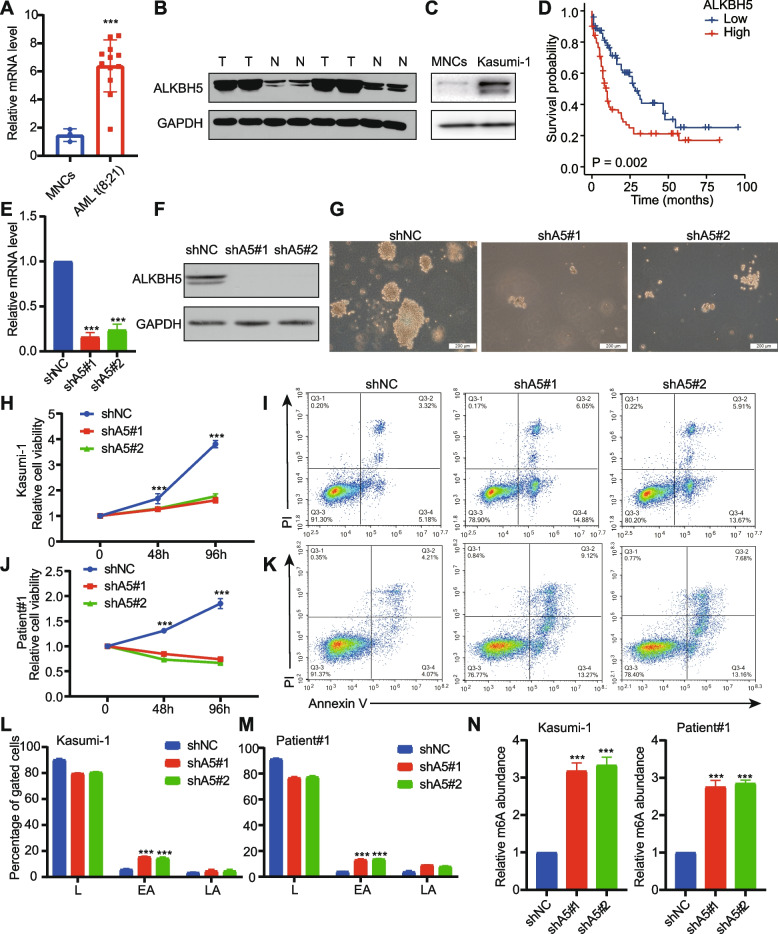
ALKBH5 is required for cell growth of t (8;21) AML. **A** Comparison of the mRNA levels of ALKBH5 in normal BMMNCs (*n* = 3) and t (8;21) AML patient BMMNCs (*n* = 12). **B** Comparison of the protein levels of ALKBH5 between normal and AML patients. **C** Western blot of ALKBH5 protein between normal MNCs and Kasumi-1 cells. **D** Kaplan–Meier curve analysis between high-ALKBH5 and low-ALKBH5 groups in TCGA-LAML cohort. The median of ALKBH5 expression was used as a cut-off value. The mRNA (**E**) and protein (**F**) level of ALKBH5 upon treatment with shNC, shALKBH5#1, or shALKBH5#2 in Kasumi-1 cells. Effects of ALKBH5 knockdown on colony-formation (**G**) cell growth/proliferation (**H**), and apoptosis (**I**) of Kasumi-1 cells. Effects of ALKBH5 knockdown on cell growth/proliferation (**J**), and apoptosis (**K**) of human primary AML cells from Patient #1. **L** Quantitative analysis for Fig. 1I. L: live cells; EA: early apoptotic cells; LA: late apoptotic cells. **M** Quantitative analysis for Fig. 1K. L: live cells; EA: early apoptotic cells; LA: late apoptotic cells. **N** The effects of ALKBH5 knockdown on global m6A abundance of Kasumi-1 cells and primary cells

ALKBH5 was highly expressed in t (8;21) AML patients compared with normal MNCs at the mRNA level (Fig. 1A). Our western blot data showed that ALKBH5 was expressed significantly higher in t (8;21) AML patients (Fig. 1B). Similarly, ALKBH5 protein levels are higher in Kasumi-1 cells compared with MNCs (Fig. 1C). Moreover, elevated ALKBH5 was associated with shorter overall survival (OS) in patients with AML based on the TCGA-LAML cohort (Fig. 1D).

To investigate the role of ALKBH5 in t (8;21) AML, the ALKBH5 knockdown cell model was constructed in Kasumi-1 cells (Fig. 1E-F). ALKBH5 knockdown caused substantial inhibition of proliferation (Fig. 1G-H), and significant induction of apoptosis (Fig. 1I and L). ALKBH5 knockdown also significantly inhibited human primary AML cell growth (Fig. 1J) and promoted apoptosis in human bulk AML cells (Fig. 1K and M). We further detected the global m^6^A level and observed that ALKBH5 knockdown induced a noticeable increase level in Kasumi-1 cells and primary cells (Fig. 1N).

### ALKBH5 knockdown impairs t (8;21) AML maintenance

**Fig. 2:**
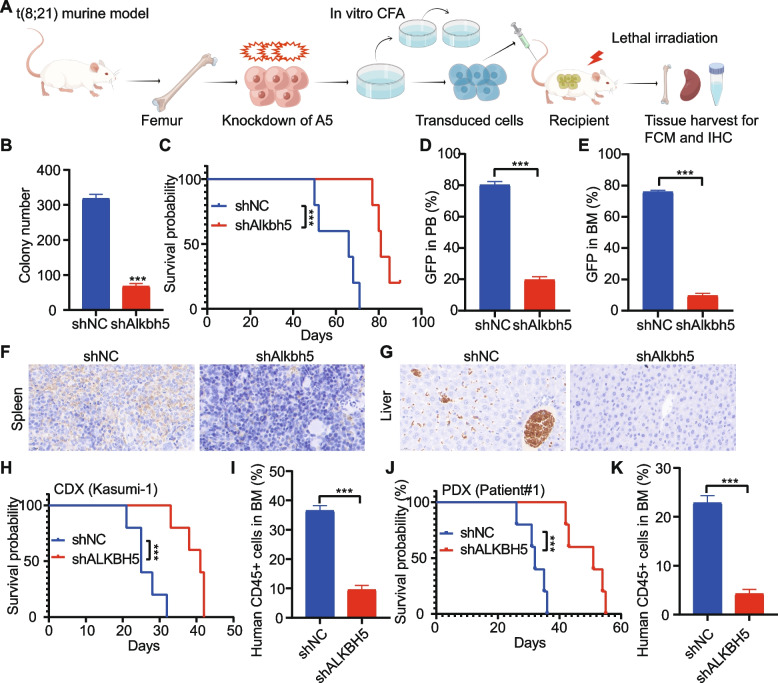
Knockdown of ALKBH5 affects the maintenance of murine and human AML. **A** Bone marrow lineage-negative (Lin^−^) cells collected from t (8;21) murine leukemia model were transduced with shNC or shAlkbh5 and used for colony-forming/replating assays (CFAs). Transduced cells were also transplanted into lethally irradiated recipient mice after the first round of colony formation for leukemogenesis. **B** Colony-forming cell counts of transduced cells. **C** Kaplan-Meier survival curves of recipient mice transplanted with transduced cells. Percentage of GFP+ cells in peripheral blood (**D**) and BM (**E**) of recipient mice. Representative IHC images of the spleen (**F**) and liver (**G**) in recipient mice transplanted with leukemic cells with shNC or shAlkbh5. **H** Kaplan-Meier survival curves of NSG mice transplanted with Kasumi-1 cells that were transduced with shNC (*n* = 5) or shALKBH5 (*n* = 5). **I** Percentage of human CD45+ cells in BM (CDX model). (J) Kaplan-Meier survival curves of NSG mice transplanted with primary t (8;21) AML cells that were transduced with shNC (*n* = 5) or shALKBH5 (n = 5). (K) Percentage of human CD45+ cells in BM (PDX model)

Firstly, RUNX1-RUNX1T1 and KIT^N822Kmut^ co-expression mice were used as a t (8;21) leukemia murine model [19]. To determine if ALKBH5 is required for t (8;21) AML maintenance, we transduced Alkbh5 shRNAs or a control shRNA into t (8;21) leukemic BM cells collected from t (8;21) leukemia mice and established BMT models (Fig. 2A). Alkbh5 knockdown dramatically inhibited colony number as detected by the in vitro colony-forming/replating assays (CFAs) (Fig. 2B). Knockdown of Alkbh5 significantly impaired the progression of RUNX1-RUNX1T1 and KIT^N822Kmut^-induced t (8;21) AML in recipient mice (Fig. 2C). We observed that Alkbh5 knockdown significantly inhibited the engraftment of transformed donor cells in peripheral blood (PB) (Fig. 2D) and BM (Fig. 2E), and reduced the infiltration of leukemic cells in the spleen (Fig. 2F) and liver (Fig. 2G). We next used the xenograft model (CDX and PDX) to further evaluate the potential role of ALKBH5 in the maintenance of human AML cells. As expected, ALKBH5 knockdown significantly prolonged survival in xenograft recipient mice and inhibited the engraftment of leukemic cells in BM (Fig. 2H-K).

### Identification of potential targets of ALKBH5 in t (8;21) AML

**Fig. 3:**
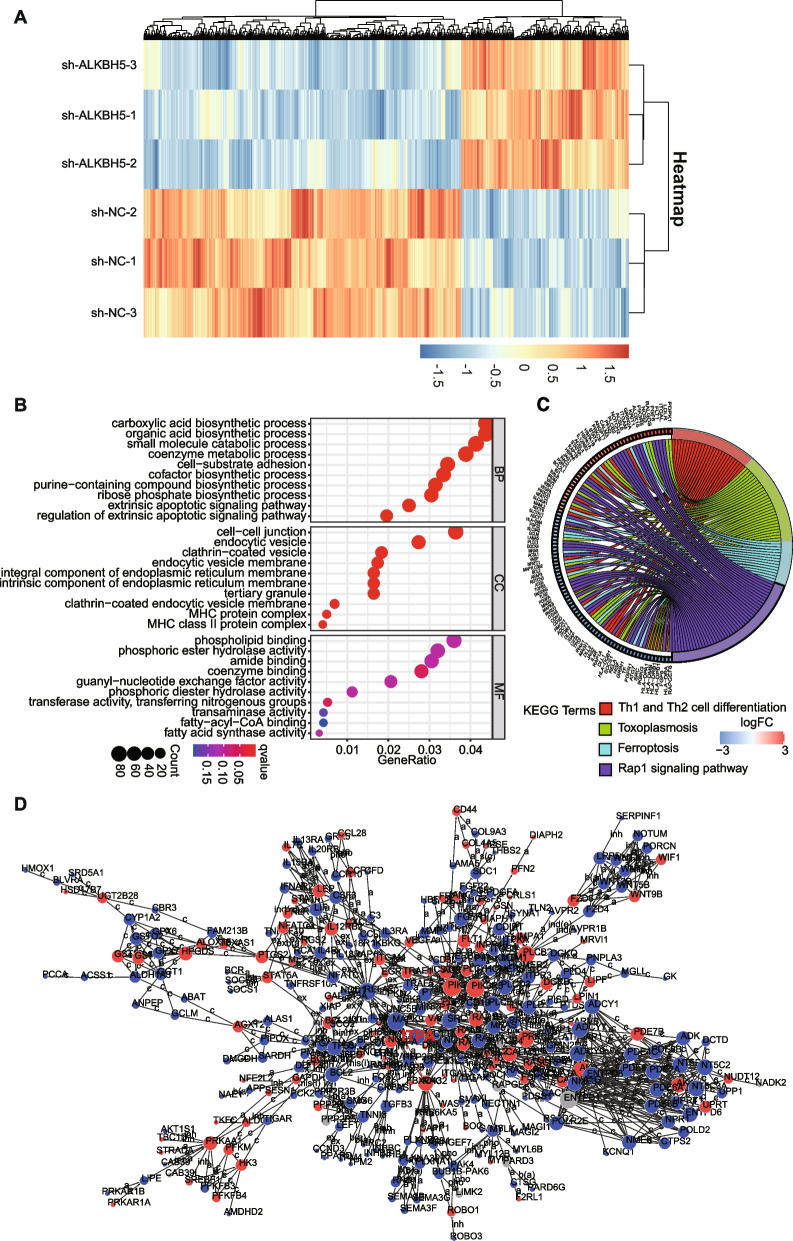
Identification of potential targets of ALKBH5 in Kasumi-1 cells. **A** The heatmap of DEGs between shNC and shALKBH5 groups in Kasumi-1 cells. The GO (**B**) and KEGG (**C**) analyses of DEGs. **D** Global signal transduction network of DEGs. Red nodes represent upregulated genes; blue nodes represent downregulated genes. The lines exhibit the interaction between genes. The size of the node indicates the degree of interaction with other genes. The larger a node, the more important a gene

To explore mechanisms underlying ALKBH5 function in t (8;21) AML, we performed transcriptome sequencing upon ALKBH5 knockdown in Kasumi-1 cells. 2326 differentially expressed genes (DEGs) were identified between 3 shALKBH5 and 3 shNC samples, including 1533 downregulated genes and 793 upregulated genes (Fig. 3A). Through Gene Ontology (GO) and Kyoto Encyclopedia of Genes and Genomes (KEGG) pathway enrichment analyses, we identified the top 10 GO terms and top 5 KEGG terms in which the apoptotic signaling pathway was included (Fig. 3B-C, Table S3-S4), which was consistent with our findings that ALKBH5 knockdown increased apoptosis in Kasumi-1 cells. We then identified key DEGs using global signal transduction network analysis (Fig. 3D). The top 10 hub genes were shown in Table S5.

### ITPA is a functionally important target of ALKBH5

After further bioinformatics analysis for the hub genes using the data from the TCGA-LAML cohort, RELA, MAPK9, and ITPA were considered positive targets of ALKBN5, whereas the other seven DEGs showed either nonsignificant or unexpected co-expression relationships with ALKBH5 (Fig. S1A-J). Among the three key targets, only ITPA showed significant value for the prediction of prognosis in AML patients based on the TCGA-LAML cohort (Fig. S2A-C). Thus, we decided to focus on ITPA for further studies.

**Fig. 4:**
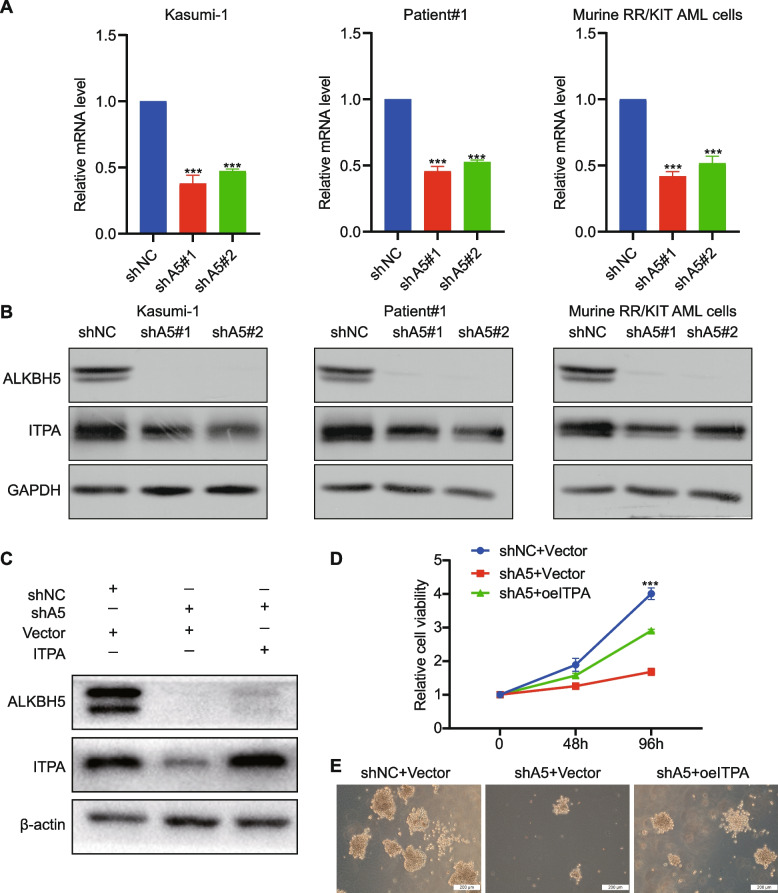
ITPA is a functionally important target of ALKBH5. **A** The mRNA level of ITPA upon ALKBH5 knockdown in Kasumi-1 cells, primary AML cells, and murine RR/KIT cells, respectively. **B** The representative WB images of ALKBH5 and ITPA upon ALKBH5 knockdown in Kasumi-1 cells, primary AML cells, and murine RR/KIT cells, respectively. **C** Western blots of ALKBH5 and ITPA in Kasumi-1 cells after transduced with shNC or shALKBH5, together with an empty (vector) or ITPA-encoding lentivirus as indicated. After drug selection, those co-transduced cells were prepared for cell proliferation assays (**D**) or colony-forming assays (**E**)

ALKBH5 knockdown significantly decreased the ITPA level in human AML cell lines, primary AML cells, and murine RR/KIT AML cells at both transcriptional (Fig. 4A) and protein levels (Fig. 4B). To investigate the functional importance of ITPA as a target of ALKBH5, we performed cell proliferation and clone formation experiments upon manipulating both ALKBH5 and ITPA expression (Fig. 4C) and found the inhibitory effects of ALKBH5 knockdown on cell growth can be largely rescued by the forced expression of ITPA (Fig. 4D-E). To determine whether the expression of ALKBH5 or ITPA was an independent prognostic factor for AML patients, we performed multivariate Cox analyses. The results showed that after adjusting clinicopathological features including age, white blood cells, and cytogenetic risk, ALKBH5 or ITPA still kept their ability to predict AML survival (Table S6 and S7).

### ALKBH5 regulates ITPA mRNA stability by altering m^6^a modification

**Fig. 5:**
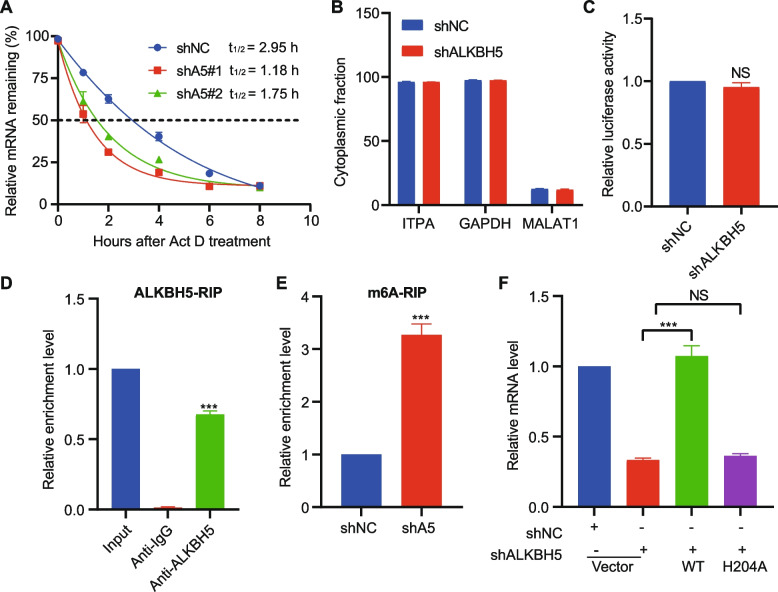
ALKBH5 regulates ITPA expression by affecting its mRNA stability. **A** The mRNA half-life (t_1/2_) of ITPA in Kasumi-1 cells upon ALKBH5 knockdown. **B** qPCR analysis of the distribution of ITPA, GAPDH mRNA, and MALAT1 RNA in subcellular fractions from Kasumi-1 cells. GAPDH mRNA is predominantly located in the cytoplasm, whereas MALAT1 RNA is predominantly located in the nucleus. **C** The promoter activity of ITPA in Kasumi-1 cells transduced with shNC or shALKBH5. **D** ALKBH5-RIP qPCR validation of ALKBH5 binding of ITPA. **E** Gene-specific m6A-RIP qPCR validation of m6A level changes of ITPA. **F** The mRNA level of ITPA in Kasumi-1 cells transduced with shNC or shALKBH5, together with an empty (vector) or wild-type ALKBH5-encoding lentivirus or the H204A mutant

Since ITPA was a positive target of ALKBH5 and a driver for the altered proliferative ability, we next investigated the regulatory mechanism of ITPA expression by ALKBH5. ALKBH5 knockdown may affect mRNA export and RNA metabolism [20], so we assessed the stability and subcellular localization of ITPA mRNA. ALKBH5 knockdown significantly decreased ITPA mRNA half-life in Kasumi-1 cells (Fig. 5A), whereas did not appear to affect the nuclear retention or export of ITPA RNA (Fig. 5B). Furthermore, ALKBH5 knockdown did not affect ITPA promoter activity as determined using luciferase reporter assays (Fig. 5C).

To ascertain whether ITPA mRNA is a substrate for ALKBH5, we used RNA immunoprecipitation (RIP) combined with a qPCR assay. The results indicated that the anti-ALKBH5 antibody could significantly enrich ITPA transcripts compared with the anti-IgG antibody (Fig. 5D). Further, we used methylated RNA immunoprecipitation (MeRIP) combined with qPCR to determine the ITPA m^6^A methylation levels following ALKBH5 knockdown. ALKBH5 knockdown increased the m^6^A level of ITPA mRNA compared with the control (Fig. 5E). Overexpression of the wild-type ALKBH5 but not the reported catalytic inactive mutant ALKBH5 H204A was able to restore ITPA expression (Figs. 5F), indicating that ALKBH5 mainly affects ITPA expression through its demethylation activity. Overall, our RIP-qPCR, and gene-specific m6A-qPCR confirmed that ITPA were strongly bound by ALKBH5 and associated with significantly increased m6A abundance and expected expression level changes in AML cells upon ALKBH5 knockdown.

### TCF15 regulates ALKBH5 expression by elevating its promoter activity

**Fig. 6:**
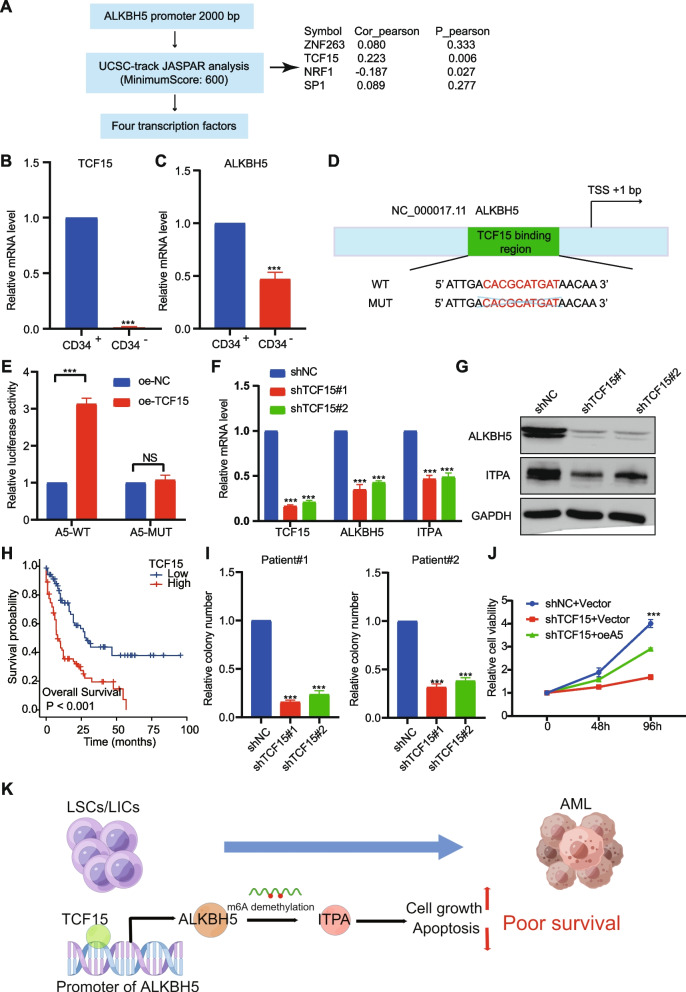
TCF15 regulates ALKBH5 expression by elevating its promoter activity. **A** Flowchart of screening for transcription factors. mRNA level of TCF15 (**B**) or ALKBH5 (**C**) in CD34^+^ LSCs/LICs and CD34^−^ bulk AML cells, respectively. **D**-**E** CD34^+^ LSCs/LICs cells were transduced with reporter plasmids containing either wild or mutant ALKBH5 promoter fragments. Then, luciferase activity was measured in CD34^+^ LSCs/LICs cells transduced with the empty vector or TCF15. qPCR (**F**) and western blots (**G**) of TCF15, ALKBH5, and ITPA in LSCs transduced with shNC, shTCF15#1, or shTCF15#2. **H** Kaplan–Meier curve analysis between high-TCF15 and low-TCF15 groups in TCGA-LAML cohort. The median of TCF15 expression was used as a cut-off value. **I** Colony-forming cell counts of transduced cells. **J** Primary cells were transduced with shNC or shTCF15, together with an empty (vector) or ALKBH5-encoding lentivirus. After drug selection, those co-transduced cells were prepared for cell proliferation assays. **K** The role of TCF15/ALKBH5/ITPA in AML progression, drawn by Figdraw

To investigate why ALKBH5 is highly expressed in AML, we focused on transcription factors enriched on the promoter of ALKBH5. We obtained the ALKBH5 promoter region (− 2000 to + 1 of TSS) from the NCBI database, and then four transcription factors were identified by the UCSC-track JASPAR analysis (criterion: Score > 600), (Fig. 6A). According to the TCGA-LAML dataset, we found TCF15 and NRF1 were significantly associated with ALKBH5 via Pearson analysis (Fig. S3A-D).

As a known transcription factor, TCF15 is essential for pluripotency exit, hematopoietic stem cells (HSCs) quiescence, and HSC long-term self-renewal [16]. However, the role of TCF15 in AML is still unclear. In AML patients, drug resistance and relapse have been linked with the existence of LSCs/LICs [17]. Since Tcf15 expression is specific to HSCs [16 21] and is required for HSC long-term self-renewal, we speculated that TCF15 was required for LSC/LIC self-renewal. TCF15 was expressed in CD34^+^ LSCs/LICs instead of CD34^−^ bulk AML cells (Fig. 6B). In addition, ALKBH5 was highly expressed in CD34^+^ LSCs/LICs compared with CD34^−^ bulk AML cells (Fig. 6C). To determine whether TCF15 directly regulated ALKBH5, we conducted luciferase reporter assays. The deletion mutation of potential binding sites abolished the transactivation of the ALHBH5 promoter by TCF15 overexpression (Fig. 6D-E). TCF15 knockdown significantly decreased the expression of ALKBH5 and ITPA at both transcription and protein levels in LSCs (Fig. 6F-G).

AML patients with higher TCF15 expression were associated with shorter OS based on the TCGA-LAML cohort (Fig. 6H). ALKBH5 knockdown significantly inhibited the colony-forming ability of human LSCs/LICs (Fig. 6I). Moreover, the inhibitory effects of TCF15 knockdown on cell growth can be largely rescued by the forced expression of ALKBH5 (Fig. 6J). Overall, the TCF15/ALKBH5/ITPA axis plays an essential role in the progression of t (8;21) AML (Fig. 6K).

## Discussion

Alkbh5 knockout mice are viable, but their spermatogenesis is compromised [20], suggesting the contribution of ALKBH5 is restricted to a subset of cells versus participating in the systemic regulation of vertebrate development. Recently, research on ALKBH5 in disease has been focused primarily on cancer. These findings suggest that ALKBH5 is frequently dysregulated in multiple malignancies, and plays a critical role as an m6A demethylase [6]. In this study, we found that ALKBH5 knockdown inhibits t (8;21) AML cell proliferation and promotes its apoptosis via targeting ITPA. In the t (8;21) LSCs, transcription factor TCF15 positively regulates ALKBH5 expression to exert its oncogenic function.

Inosine triphosphatase (ITPA), encoded by the ITPA gene, plays a vital role in cell growth and DNA stability [21, 22]. Maintaining the purity of the nucleotide pool ensures precise DNA replication and transcription. ITPA can prevent the incorporation of noncanonical purine nucleotides into DNA and RNA to maintain normal DNA metabolism [23]. Since cancer cells replicate DNA at a faster rate than normal cells [24], cancer may be more hypersensitive to ITPA malfunctions. Silencing of ITPA mediated by nano-carrier was reported to promote apoptosis in human breast cancer cells, which provides promise for targeted therapeutic application [23]. ITPA deficiency induces cell growth delay via upregulating P21 expression in human colorectal cancer [21]. In AML, transcriptional up-regulation of ITPA was noted in MLL-amplified samples that were associated with adverse prognosis [25]. We found that ITPA was a functionally important target of ALKBH5 in t (8;21) leukemia cells. Forced expression of ITPA can rescue the growth inhibitory effects of ALKBH5 knockdown.

The reversible m6A modification plays a role in multiple pathways controlling mRNA metabolism and gene regulation. Indeed, m6A modulates mRNA splicing [26], enhances nuclear export of mRNAs [27], alters mRNA stability [28], increases translation efficiency [29], and facilitates noncanonical translation initiation [30]. We found that ALKBH5 knockdown significantly decreased ITPA mRNA half-life in Kasumi-1 cells while showing no effect on the export of ITPA mRNA or the promoter activity of ITPA. In all biological systems, RNA is a central mechanism for information transmission, and there are a number of opportunities for developing small-molecule therapeutics that target it [31]. Protein gene products could be modulated by altering translation efficiency, abundance, or stability of mRNAs if mRNAs could be targeted. Moreover, modulating mRNAs before or during protein biogenesis may enable drugs to target proteins that are difficult to target or undruggable. Hence, uncovering the mechanism of ALKBH5 modulating the mRNA metabolism of ITPA may contribute to the AML treatment.

There has been an increase in research into the mechanisms of ALKBH5 dysregulation in cancer. These findings indicated that ALKBH5 dysregulation in cancer is primarily caused by hypoxia, epigenetic modulators, transcription factors, and non-coding RNAs [6]. As transcription factors bind DNA in a sequence-specific manner, they control chromatin and transcription, forming a complicated system that affects gene expression [32]. Through UCSC-track JASPAR analysis (criterion: Score > 600), we obtained 4 candidate genes, including ZNF263, TCF15, NRF1, and SP1, among which only TCF15 is significantly associated with ALKBH5. We confirmed that TCF15 knockdown could impair the proliferation ability of LSCs and forced expression of ALKBH5 can partially rescue the role of TCF15 knockdown in LSCs.

In conclusion, we found that the TCF15/ALKBH5/ITPA axis plays a vital role in AML pathogenesis and LSC/LIC maintenance. AML with t (8;21) has a good prognosis and most patients enter remission, however, approximately half of the patient’s relapse, and only 60% of the patients live beyond 5 years after diagnosis. In the future, eliminating LSCs/LICs by targeting TCF15/ALKBH5/ITPA represents a promising therapeutic strategy for the treatment of t (8;21) AML patients.

## Supplementary Information

**Additional file 1: Fig. S1.** The association between top 10 key DEGs and ALKBH5 expression.

**Additional file 2: Fig. S2.** The Kaplan–Meier curve analysis for ITPA, RELA, and MAPK9 in TCGA-LAML cohort. The median of gene expression was used as a cut-off value.

**Additional file 3: Fig. S3.** The relationship between transcription factors and ALKBH5 expression.

**Additional file 4: Table S1.** Patient characteristics. **Table S2.** The information of primers. **Table S3.** The results of GO analyses. **Table S4.** The results of KEGG analysis. **Table S5.** The results of global signal transduction network analysis. **Table S6.** The univariate and multivariate analysis of AML clinical factors and ALKBH5 expression. **Table S7.** The univariate and multivariate analysis of AML clinical factors and ITPA expression.

## Data Availability

RNA-seq data used in this study have been deposited in the National Center for Biotechnology Information Sequence Red Archive (SRA) under the accession code SRR17828461-SRR17828472.
