# Supplementary figures and images for: RNA demethylase ALKBH5 promotes tumorigenesis of t (8;21) acute myeloid leukemia via ITPA m6A modification
Source: Biomark Res. 2023 Mar 10;11:30. doi: 10.1186/s40364-023-00464-x (PMC10007764; doi:10.1186/s40364-023-00464-x)

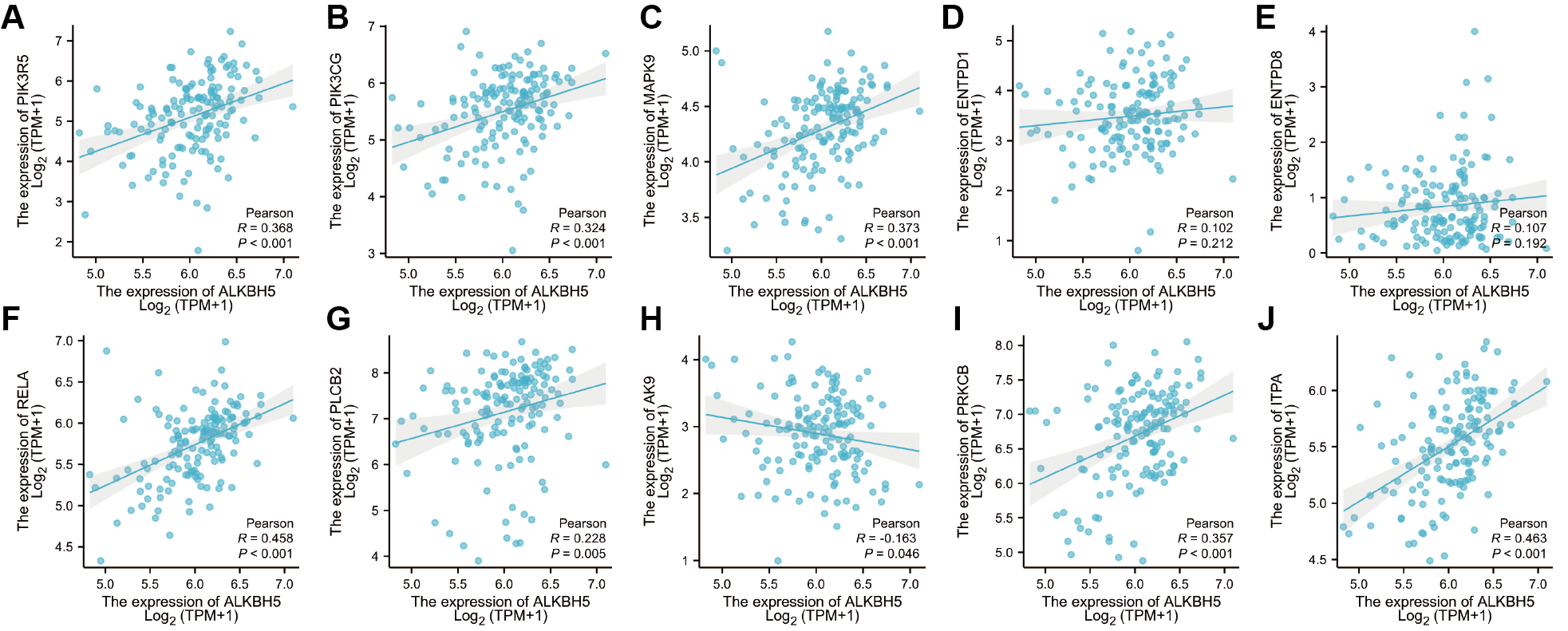

Supplement: Supplementary file 1 — Additional file 1: Fig. S1. The association between top 10 key DEGs and ALKBH5 expression. [file 40364_2023_464_MOESM1_ESM.tif]

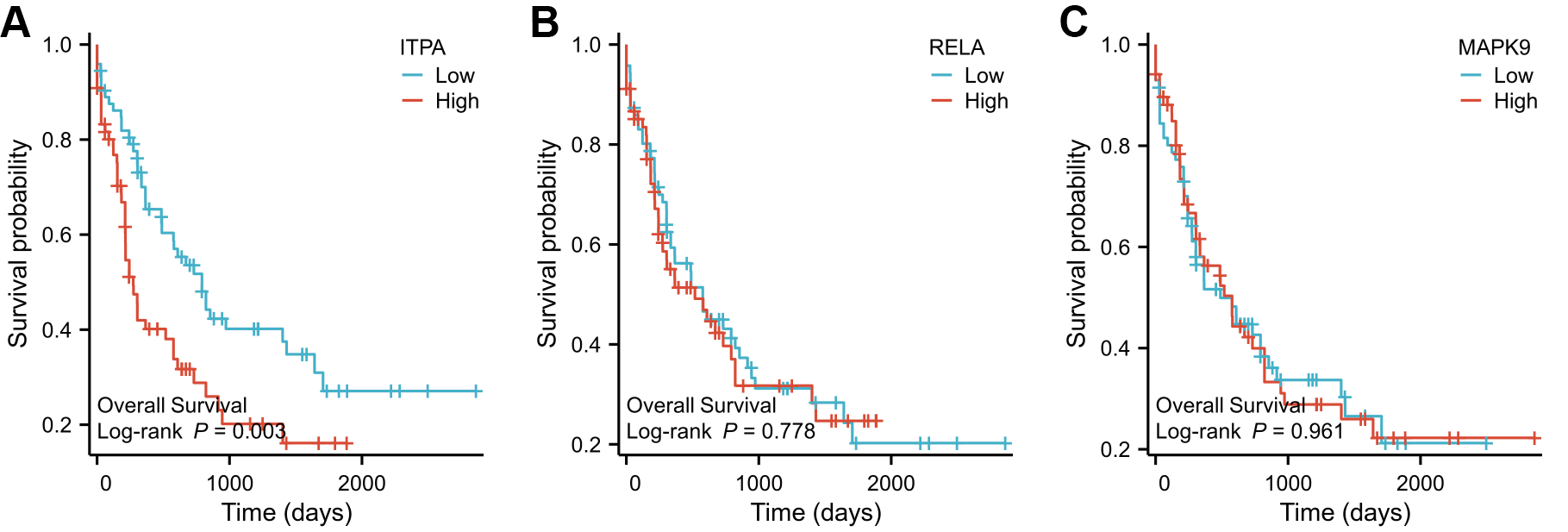

Supplement: Supplementary file 2 — Additional file 2: Fig. S2. The Kaplan–Meier curve analysis for ITPA, RELA, and MAPK9 in TCGA-LAML cohort. The median of gene expression was used as a cut-off value. [file 40364_2023_464_MOESM2_ESM.tif]

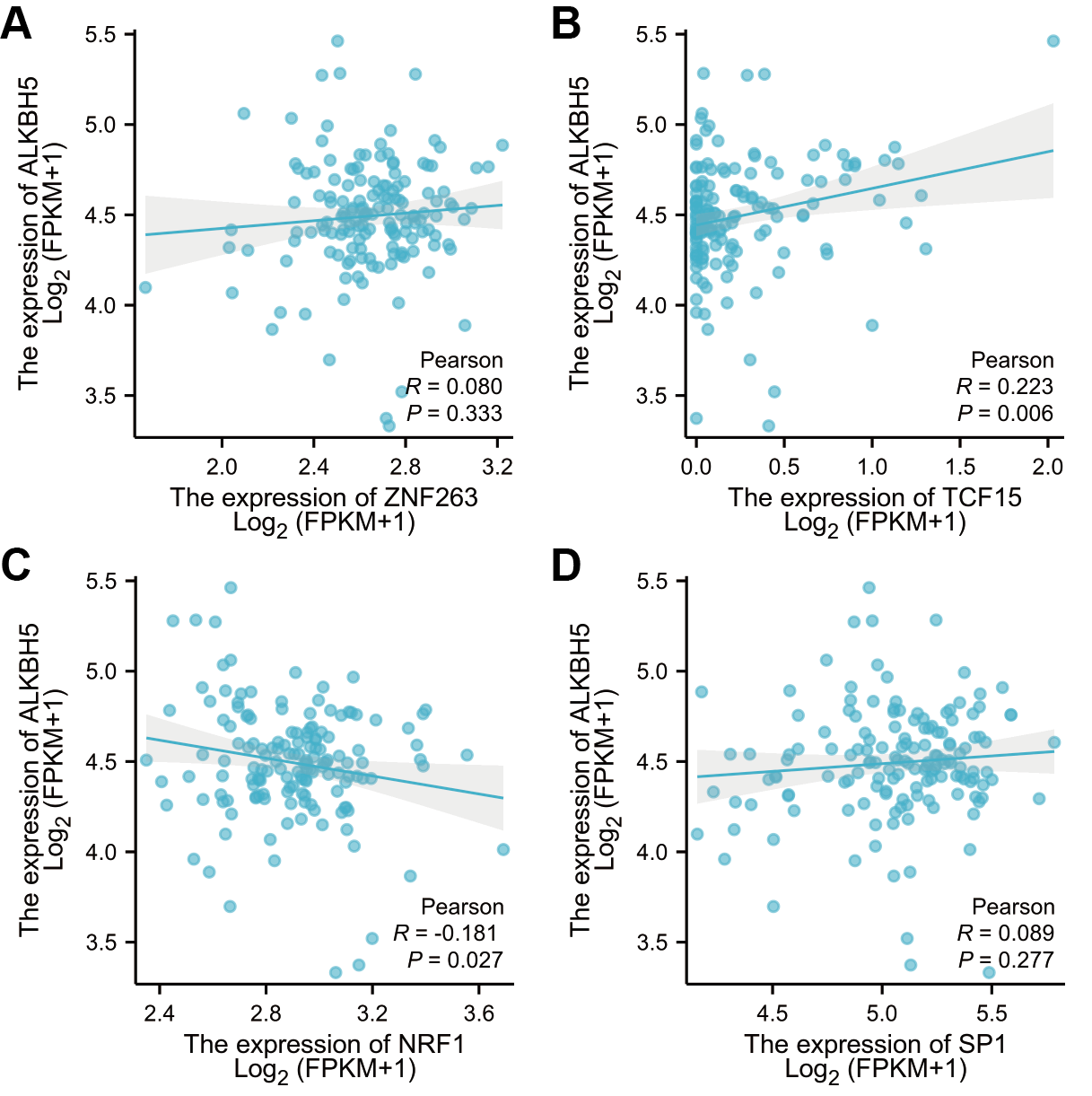

Supplement: Supplementary file 3 — Additional file 3: Fig. S3. The relationship between transcription factors and ALKBH5 expression. [file 40364_2023_464_MOESM3_ESM.tif]
